# Sparse Array Angle Estimation Using Reduced-Dimension ESPRIT-MUSIC in MIMO Radar

**DOI:** 10.1155/2013/784267

**Published:** 2013-11-24

**Authors:** Chaozhu Zhang, Yucai Pang

**Affiliations:** College of Information and Communication Engineering, Harbin Engineering University, Harbin 150001, China

## Abstract

Sparse linear arrays provide better performance than the filled linear arrays in terms of angle estimation and resolution with reduced size and low cost. However, they are subject to manifold ambiguity. In this paper, both the transmit array and receive array are sparse linear arrays in the bistatic MIMO radar. Firstly, we present an ESPRIT-MUSIC method in which ESPRIT algorithm is used to obtain ambiguous angle estimates. The disambiguation algorithm uses MUSIC-based procedure to identify the true direction cosine estimate from a set of ambiguous candidate estimates. The paired transmit angle and receive angle can be estimated and the manifold ambiguity can be solved. However, the proposed algorithm has high computational complexity due to the requirement of two-dimension search. Further, the Reduced-Dimension ESPRIT-MUSIC (RD-ESPRIT-MUSIC) is proposed to reduce the complexity of the algorithm. And the RD-ESPRIT-MUSIC only demands one-dimension search. Simulation results demonstrate the effectiveness of the method.

## 1. Introduction

MIMO radar employs multiple transmit and receive elements and has the ability to jointly plan transmissions and process received signals. It has recently been the focus of research owing to its significant performance improvement compared to the conventional phased-array radar [1–3]. MIMO radar has more degrees of freedom than other systems with a single transmit element. These additional degrees of freedom are well-qualified for overcoming fading effect, enhancing spatial resolution, strengthening parameter identifiability, and also improving target detection performance [4–6].

In order to estimate the transmit angle and receive angle in the bistatic MIMO radar, some methods have been proposed [7, 8]. However, both of them cannot be applied in sparse linear arrays. In [9], the authors discuss the imaging method of the moving targets in MIMO radar with sparse array. Also, the issue of sparsity in the specific context of a MIMO radar system is studied in [10]. Sparse linear arrays can provide a large measurement basis with a reduced number of elements as compared to filled arrays. But the disadvantage of sparse array is manifold ambiguity which can cause large angle estimation errors. In [11, 12] the authors discuss the angle estimation performance for different sparse linear array configurations using the Ziv-Zakai bound (ZZB). They illustrate that the ZZB includes three terms which correspond to the three types of estimation errors: random errors, small main lobe errors, and errors due to side lobe ambiguities. 

In conventional radar, three types of methods are proposed to solve the ambiguity problem in angle estimation. The first type is to change the original array by inducing an additional sensor or sliding the sensors positions [13–15]. The second type is to optimize the sensor positions of an array [16–18]. The last type focuses on the algorithms rather than on the hardware [19–21].

In this paper, we present an angle estimation method based on ESPRIT and MUSIC to avoid the manifold ambiguity for the bistatic MIMO radar spaced sparse linear arrays. The main idea of the method uses MUSIC-based procedure to search the true direction cosine estimate from a set of ambiguous candidate estimates. And the ambiguous candidate estimates are obtained by ESPRIT algorithm. But, the requirement of two-dimension search renders much higher computational cost. Then we derive a Reduced-Dimension ESPRIT-MUSIC algorithm which reduces the complexity for angle estimation in the bistatic MIMO radar with sparse linear arrays. Compared to the uniform linear array (ULA), we can obtain more accurate angle estimation.

## 2. Problem Formulation

**Figure 1:**
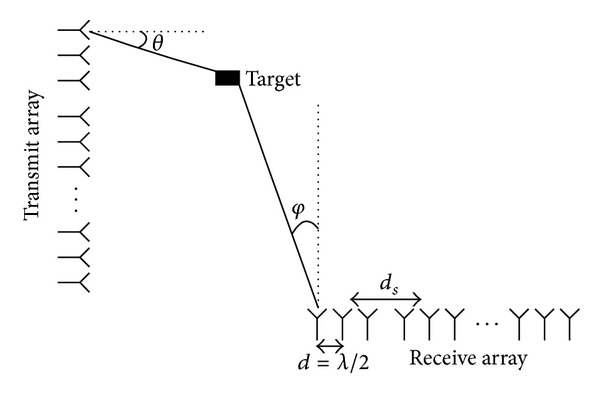
Bistatic MIMO radar with sparse linear arrays.

As shown in Figure 1, a bistatic MIMO radar system consists of *M*_*t*_-element transmit array and *M*_*r*_-element receive array, both of which are spaced sparse linear arrays. The transmit array is composed of *N*_*st*_ subarrays with *M*_*st*_ sensors per subarray, and the receive array is composed of *N*_*sr*_ subarrays with *M*_*sr*_ sensors per subarray, intersensor spacing *d* = *λ*/2 and intersubarray spacing *d*_*s*_ ≫ *d*. The targets are assumed to be in the far-field of transmit and receive arrays. At the transmit site, *M*_*t*_ different narrowband signals are emitted simultaneously, which have identical bandwidth and centre frequency but are temporally orthogonal. Assume that *P* noncoherent targets, respectively, correspond to the transmit angle *θ*_*p*_ and the receive angle *φ*_*p*_, where *p* = 1,…, *P*, are presented in the same range. For convenience, we consider that estimation of angle cosines are relative to the array axis *μ*_*p*_ = sin*θ*_*p*_, *ν*_*p*_ = sin*φ*_*p*_.

The output of the entire matched filters at the receiver can be expressed as

(1) $$\begin{array}{c} x\left(t\right)=As\left(t\right)+n\left(t\right), \end{array}$$

where **A** = [**a**_1_, **a**_2_,…, **a**_*P*_] is an *M*_*t*_ *M*_*r*_ × *P* matrix composed of the *P* steering vectors and **a**_*p*_ = **a**(*ν*_*p*_) ⊗ **a**(*μ*_*p*_) is the Kronecker product of the receive and the transmit steering vectors for the *p*th target. 

And the transmit steering vectors can be written as

(2) $$\begin{array}{c} a\left(\mu_{p}\right)=a_{s}\left(\mu_{p}\right)⊗a_{l}\left(\mu_{p}\right), \end{array}$$

where **a**_*l*_(*μ*_*p*_) is the steering vector of the transmit subarray which is defined by

(3) $$\begin{array}{c} a_{l}\left(\mu_{p}\right)={\left[1,e^{j\omega_{tp}},\ldots ,e^{j(M_{st}-1)\omega_{tp}}\right]}^{T}, \end{array}$$

where *ω*_*tp*_ = (2*π*/*λ*)*dμ*_*p*_, (·)^*T*^ stands for transpose and **a**_*s*_(*μ*_*p*_) is the vector describing the phase shifts caused by transmit subarray displacements

(4) $$\begin{array}{c} a_{s}\left(\mu_{p}\right)={\left[1,e^{j\omega_{tp}^{a}},\ldots ,e^{j(N_{st}-1)\omega_{tp}^{a}}\right]}^{T}, \end{array}$$

where *ω*_*tp*_^*a*^ = (2*π*/*λ*)*d*_*s*_ *μ*_*p*_.

Also, the receive steering vectors can be expressed as

(5) $$\begin{array}{c} a\left(\nu_{p}\right)=a_{s}\left(\nu_{p}\right)⊗a_{l}\left(\nu_{p}\right), \end{array}$$

where **a**_*l*_(*ν*_*p*_) is the steering vector of the receive subarray which is defined by

(6) $$\begin{array}{c} a_{l}\left(\nu_{p}\right)={\left[1,e^{j\omega_{rp}},\ldots ,e^{j(M_{sr}-1)\omega_{rp}}\right]}^{T}, \end{array}$$

where *ω*_*rp*_ = (2*π*/*λ*)*dν*_*p*_ and **a**_*s*_(*ν*_*p*_) is the vector describing the phase shifts caused by receive subarray displacements

(7) $$\begin{array}{c} a_{s}\left(\nu_{p}\right)={\left[1,e^{j\omega_{rp}^{a}},\ldots ,e^{j(N_{sr}-1)\omega_{rp}^{a}}\right]}^{T}, \end{array}$$

where *ω*_*rp*_^*a*^ = (2*π*/*λ*)*d*_*s*_ *ν*_*p*_.

**s**(*t*) = [*s*_1_(*t*), *s*_2_(*t*),…, *s*_*P*_(*t*)]^*T*^ is a column vector consisting of the phase and amplitudes of the *P* sources at time *t* which is usually in the form of *s*_*p*_ = *α*_*p*_ *e* ^*jω*_*dp*_*t*^ with *ω*_*dp*_ being the Doppler frequency and *α*_*p*_ the amplitude involving the reflection coefficients and path losses and so on. The *M*_*t*_ *M*_*r*_ × 1 noise vector **n**(*t*) is assumed to be independent, zero-mean complex Gaussian distribution. 

## 3. ESPRIT-MUSIC for Angle Estimation

### 3.1. The Theory of ESPRIT-MUSIC Algorithm

The maximum likelihood (ML) estimation of the covariance of **x**(*t*) for *L* snapshots is **R** = 1/*L*∑_*l*=1_^*L*^ **x**(*t*_*l*_)**x** ^*H*^(*t*_*l*_), where [·]^*H*^ denotes the Hermitian transpose. Let **E**_*s*_ be the *M*_*t*_ *M*_*r*_ × *P* signal subspace matrix composed of the *P* eigenvectors corresponding to the largest *P* eigenvalues of **R**, and the last *M*_*t*_ *M*_*r*_ − *P* eigenvectors constitute the noise subspace **E**_*n*_. It can be shown that **A** and **E**_*s*_ span the same subspace. Therefore, there exists a unique nonsingular **T** such that **E**_*s*_ = **A** **T**. We define a new *M*_*t*_ *M*_*r*_ × *P* matrix **A**′ = [**a**_1_′, **a**_2_′,…, **a**_*P*_′], where **a**_*P*_′ = **a**(*μ*_*p*_) ⊗ **a**(*ν*_*p*_). Then the matrix **A**′ is row equivalent to **A**. **E**_*s*_′ is an *M*_*t*_ *M*_*r*_ × *P* signal subspace matrix formed from **E**_*s*_ by the same row interchange operations as **A**′ is formed from **A**. Let **A**_*r*1_ and **A**_*r*2_ be the *M*_*st*_ *N*_*st*_ *M*_*sr*_(*N*_*sr*_ − 1) × *P* submatrices of **A** consisting of the first and the last *M*_*st*_ *N*_*st*_ *M*_*sr*_(*N*_*sr*_ − 1) rows of **A**, respectively. Then **A**_*r*2_ = **A**_*r*1_Φ_*r*_, where Φ_*r*_ = diag⁡[*e* ^*jω*_*r*1_^*a*^^, *e* ^*jω*_*r*2_^*a*^^,…, *e* ^*jω*_*rP*_^*a*^^]. Here diag⁡(**b**) denotes a diagonal matrix constructed by the vector **b**. Let **E**_*r*1_ and **E**_*r*2_ be the *M*_*st*_ *N*_*st*_ *M*_*sr*_(*N*_*sr*_ − 1) × *P* submatrices formed from **E**_*s*_ in the same way as the **A**_*r*1_ and **A**_*r*2_ are formed from **A**. Then the diagonal elements of Φ_*r*_ are the eigenvalues of Ψ_*r*_ = **T** ^−1^Φ_*r*_ **T**, which satisfy **E**_*r*2_ = **E**_*r*1_Ψ_*r*_. The eigenvalue decomposition of Ψ_*r*_ yields ${\hat{\Phi }}_{r}=Q^{-1}\Psi_{r}Q$, where ${\hat{\Phi }}_{r}$ is a diagonal matrix composed of the eigenvalues of Ψ_*r*_ and the columns of **Q** are eigenvectors of Ψ_*r*_. Thus, **A**′ = **E**_*s*_′**Q** [8]. Similarly, let **A**_*t*1_ and **A**_*t*2_ be the submatrices *M*_*st*_(*N*_*st*_ − 1)*M*_*sr*_ *N*_*sr*_ × *P* of **A**′ consisting of the first and the last *M*_*st*_(*N*_*st*_ − 1)*M*_*sr*_ *N*_*sr*_ rows of **A**′. Then **A**_*t*2_ = **A**_*t*1_Φ_*t*_, where Φ_*t*_ = diag⁡[*e* ^*jω*_*t*1_^*a*^^, *e* ^*jω*_*t*2_^*a*^^,…, *e* ^*jω*_*tP*_^*a*^^]. So we obtain ${\hat{\xi }}_{tp}^{a}=\exp(j\omega_{tp}^{a})$, ${\hat{\xi }}_{rp}^{a}=\exp(j\omega_{rp}^{a})$, and the set of low-variance but cyclically ambiguous direction cosine estimates

(8) $$\begin{array}{c} {\hat{\mu }}_{p}^{a}=\frac{\arg\left({\hat{\xi }}_{tp}^{a}\right)}{\left(2\pi d_{s}/\lambda \right)},\\ {\hat{\nu }}_{p}^{a}=\frac{\arg\left({\hat{\xi }}_{rp}^{a}\right)}{\left(2\pi d_{s}/\lambda \right)}. \end{array}$$

Because *d*_*s*_ ≫ *d*, here exists a set of cyclically related candidates for the low-variance estimate of *μ*_*p*_ = sin*θ*_*p*_, *υ*_*p*_ = sin*φ*_*p*_, *p* = 1,…, *P*. It can be written as

(9) $$\begin{array}{c} \mu_{p}^{\left(l\right)}=\mu_{p}^{a}+l_{\mu }\left(\frac{\lambda }{d_{s}}\right),\\ \nu_{p}^{\left(l\right)}=\nu_{p}^{a}+l_{\nu }\left(\frac{\lambda }{d_{s}}\right); \end{array}$$

we define $l_{1}=(d_{s}/\lambda )(-1-{\hat{\mu }}_{p}^{a})$, $l_{2}=(d_{s}/\lambda )(1-{\hat{\mu }}_{p}^{a})$, $l_{3}=\left(d_{s}/\lambda \right)\left(-1-{\hat{\nu }}_{p}^{a}\right)$, and $l_{4}=(d_{s}/\lambda )(1-{\hat{\nu }}_{p}^{a})$, the bounds for parameter *l*_*μ*_, *l*_*ν*_ are determined by

(10) $$\begin{array}{c} \left\lceil l_{1}\right\rceil \leq l_{\mu }\leq \left\lfloor l_{2}\right\rfloor ,\\ \left\lceil l_{3}\right\rceil \leq l_{\nu }\leq \left\lfloor l_{4}\right\rfloor , \end{array}$$

where ⌈*z*⌉ denotes the smallest integer greater than *z* and ⌊*z*⌋ denotes the largest integer less than *z*. The unambiguous estimates of direction cosines can be obtained based on the 2D-MUSIC null spectrum

(11) (μ^pd,ν^pd)=argminμp(l),νp(l)[apHEnEnHap]=argminμp(l),νp(l)[(a(νp)⊗a(μp))HEnEnH(a(νp)⊗a(μp))].

Then the paired transmit angle *θ*_*p*_ and receive angle *φ*_*p*_ can be written as ${\hat{\theta }}_{p}=a\sin({\hat{\mu }}_{p}^{d})*180/\pi$, ${\hat{\varphi }}_{p}=a\sin({\hat{\nu }}_{p}^{d})*180/\pi$.

The major steps of ESPRIT-MUSIC algorithm for angle estimation in the bistatic MIMO radar with sparse linear arrays are as follows.

*Step  1.* Perform eigen-decomposition operation for the covariance matrix $\hat{R}$ to obtain **E**_*s*_ and **E**_*n*_.

*Step  2.* Calculate the ambiguous angle cosine estimates ${\hat{\mu }}_{p}^{a}$ and ${\hat{\nu }}_{p}^{a}$ via (8).

*Step  3.* Find the set of cyclically related candidates *μ*_*p*_^(*l*)^ and *ν*_*p*_^(*l*)^ by (9).

*Step  4.* Searching *μ*_*p*_ ∈ *μ*_*p*_^(*l*)^, *ν*_*p*_ ∈ *ν*_*p*_^(*l*)^ with respect to (11), the *P* largest peaks $\left[({\hat{\mu }}_{1}^{d},{\hat{\nu }}_{1}^{d}),({\hat{\mu }}_{2}^{d},{\hat{\nu }}_{2}^{d}),\ldots ,({\hat{\mu }}_{P}^{d},{\hat{\nu }}_{P}^{d})\right]$ can be found. Then compute the transmit angle *θ*_*p*_ and the receive angle *φ*_*p*_.

### 3.2. Simulation Results

**Figure 2:**
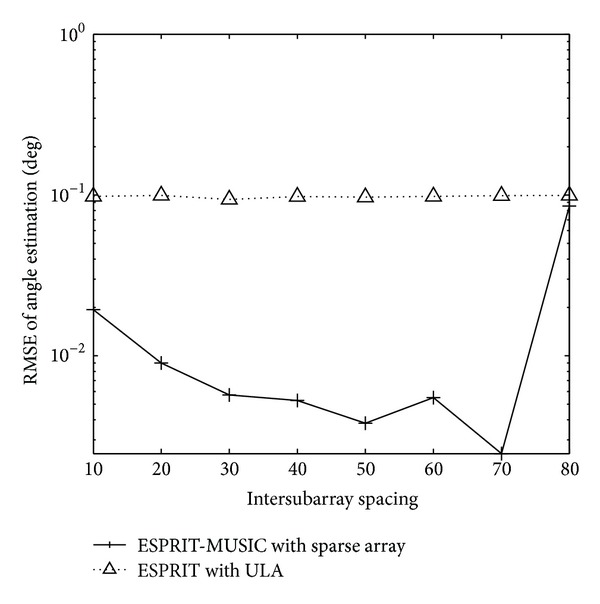
RMSE of angle estimation of the first target with different intersubarray spacing with SNR = 4 dB.

**Figure 3:**
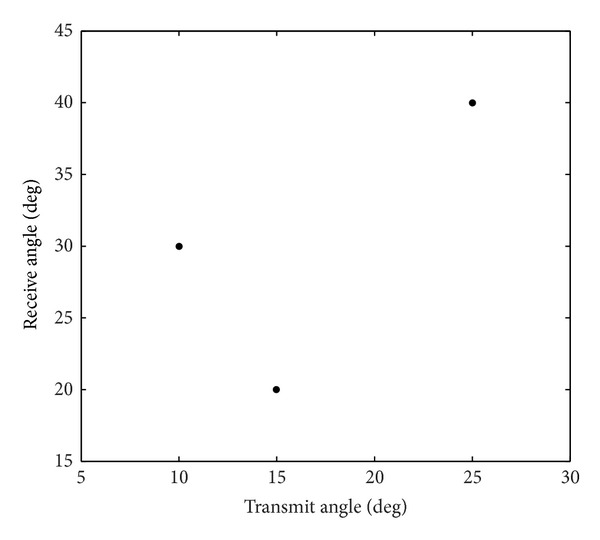
Paired results of proposed method for all three targets with SNR = 0 dB.

**Figure 4:**
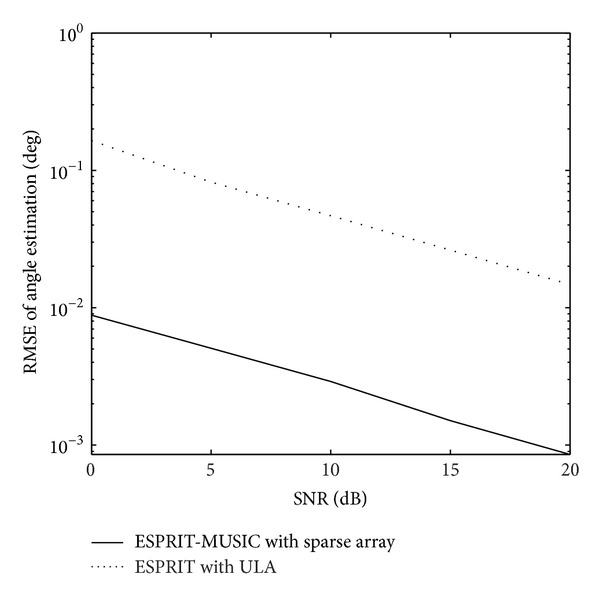
RMSE of angle estimation of the first target.

**Figure 5:**
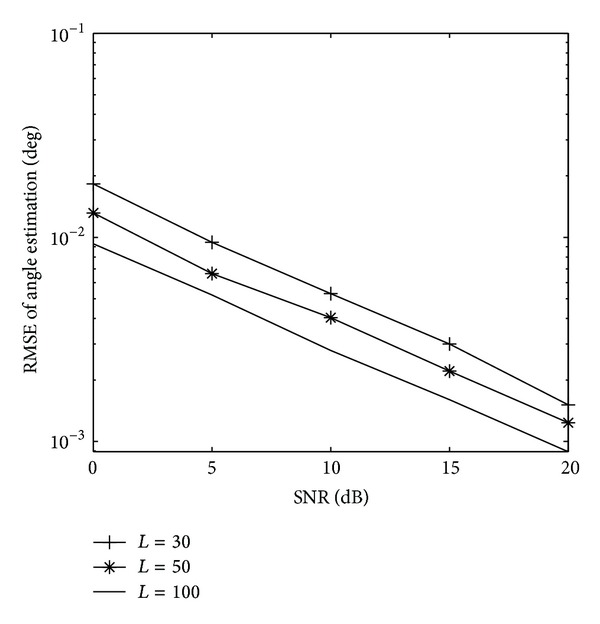
RMSE of angle estimation of the first target with different *L*.

We present 200 Monte Carlo simulations to demonstrate the angle estimation performance of ESPRIT-MUSIC algorithm. Assume that there exist *P* = 3 uncorrelated stationary targets, which are located at angles (*θ*_1_, *φ*_1_) = (15°, 20°), (*θ*_2_, *φ*_2_) = (25°, 40°), and (*θ*_3_, *φ*_3_) = (10°, 30°), and the number of snapshots is *L* for an *M*_*t*_ = 12, *M*_*st*_ = 3, *N*_*st*_ = 4 and *M*_*r*_ = 10, *M*_*sr*_ = 2, *N*_*sr*_ = 5 bistatic MIMO radar. The root mean squared error (RMSE) of the target angle estimation is defined as $\text{RMSE}=\sqrt{E{\left({\hat{\theta }}_{i}-\theta \right)}^{2}+E{\left({\hat{\varphi }}_{i}-\varphi \right)}^{2}}$, where ${\hat{\theta }}_{i}$ and ${\hat{\varphi }}_{i}$ are the estimated transmit angle and the estimated receive angle of the same target for the *i*th-trial.Figure 2 shows the RMSE of angle estimation of the first target with different intersubarray spacing, *L* = 100,  SNR = 4 dB. The angle estimation performance significant degradation in 80 half-wavelengths can be seen. So, we select the intersubarray spacing *d*_*s*_ = 30∗(*λ*/2) in the following simulation.  Figure 3 presents the paired angle estimation results of ESPRIT-MUSIC algorithm for all three targets with SNR = 0 dB,  *L* = 100. The variation of angle estimation RMSE of the proposed algorithm and the method of [8] with SNR are shown in Figure 4. From Figures 3 and 4, we can see that the paired transmit angle and receive angle are correct, and the performances of ESPRIT-MUSIC with sparse array are much better than ESPRIT with ULA.  Figure 5 depicts the algorithmic performance with different *L*. It illustrates that the angle estimation performance becomes better with *L* increasing. From Figure 5, we also draw a conclusion that the proposed algorithm has a good performance with small sampling sizes, *L* = 30.

## 4. RD-ESPRIT-MUSIC for Angle Estimation

### 4.1. The Theory of RD-ESPRIT-MUSIC Algorithm

ESPRIT-MUSIC algorithm requires two-dimension search leading to the high computational complexity. In order to reduce the computational cost of the algorithm, we propose the RD-ESPRIT-MUSIC algorithm.

In previous section, we construct the 2D-MUSIC null spectrum. And (11) is also denoted by

(12) (μ^pd,ν^pd)=argminμp(l),νp(l)[a(μp)H(a(νp)⊗IMt)HEnEnH     ×(a(νp)⊗IMt)a(μp)]=argminμp(l),νp(l)[a(μp)HB(νp)a(μp)],

where **B**(*ν*_*p*_) = (**a**(*ν*_*p*_) ⊗ **I**_*M*_*t*__)^*H*^ **E**_*n*_ **E**_*n*_^*H*^(**a**(*ν*_*p*_) ⊗ **I**_*M*_*t*__). Equation (12) is the problem of quadratic optimization. In order to eliminate the trivial solution **a**(*μ*_*p*_) = 0_*M*_*t*__, we add the constraint of **e**_1_^*H*^ **a**(*μ*_*p*_) = 1, where **e**_1_ = [1,0,…, 0]^*T*^ ∈ *R* ^*M*_*t*_×1^. The optimization problem can be reconstructed as follows:

(13) $$\begin{array}{c} \underset{\mu_{p}^{\left(l\right)},\nu_{p}^{\left(l\right)}}{\text{argmin}}\left[a{\left(\mu_{p}\right)}^{H}B\left(\nu_{p}\right)a\left(\mu_{p}\right)\right]\;,\;\text{s}.\text{t}.\;e_{1}^{H}a\left(\mu_{p}\right)=1. \end{array}$$

Then we construct the following costing function

(14) $$\begin{array}{c} L\left(\mu_{p},\nu_{p}\right)=a{\left(\mu_{p}\right)}^{H}B\left(\nu_{p}\right)a\left(\mu_{p}\right)-\lambda \left(e_{1}^{H}a\left(\mu_{p}\right)-1\right), \end{array}$$

where *λ* is constant. We have

(15) $$\begin{array}{c} \frac{\partial }{\partial a\left(\mu_{p}\right)}L\left(\mu_{p},\nu_{p}\right)=2B\left(\nu_{p}\right)a\left(\mu_{p}\right)-\lambda e_{1}=0. \end{array}$$

According to (15), **a**(*μ*_*p*_) = *μ* **B** ^−1^(*ν*_*p*_)**e**_1_, where *μ* is constant. For **e**_1_^*H*^ **a**(*μ*_*p*_) = 1, *μ* = 1/(**e**_1_^*H*^ **B** ^−1^(*ν*_*p*_)**e**_1_). So **a**(*μ*_*p*_) is obtained by

(16) $$\begin{array}{c} a\left(\mu_{p}\right)=\frac{B^{-1}\left(\nu_{p}\right)e_{1}}{e_{1}^{H}B^{-1}\left(\nu_{p}\right)e_{1}}. \end{array}$$

Inserting **a**(*μ*_*p*_) of (16) into (12), then the receive angle cosine estimates are obtained by

(17) $$\begin{array}{c} {\hat{\nu }}_{p}^{d}=\underset{\nu_{p}^{\left(l\right)}}{\text{argmin}}\frac{1}{e_{1}^{H}B^{-1}\left(\nu_{p}\right)e_{1}}=\underset{\nu_{p}^{\left(l\right)}}{\text{argmax}}\left[e_{1}^{H}B^{-1}\left(\nu_{p}\right)e_{1}\right]. \end{array}$$

Searching *ν*_*p*_ ∈ *ν*_*p*_^(*l*)^, we can find the *P* largest peaks $\left({\hat{\nu }}_{1}^{d},{\hat{\nu }}_{2}^{d},\ldots ,{\hat{\nu }}_{P}^{d}\right)$ which correspond to the receive angle. Then the receive angle *φ*_*p*_ can be obtained by ${\hat{\varphi }}_{p}=a\sin({\hat{\nu }}_{p}^{d})*180/\pi$. Also, we can obtain the *P* receive steering vectors $\left[\hat{a}(\nu_{1}),\hat{a}(\nu_{2}),\ldots ,\hat{a}(\nu_{P})\right]$. Inserting the receive steering vectors into **B**(*ν*_*p*_) = (**a**(*ν*_*p*_) ⊗ **I**_*M*_)^*H*^ **E**_*n*_ **E**_*n*_^*H*^(**a**(*ν*_*p*_) ⊗ **I**_*M*_), we obtain $\left[\hat{B}(\nu_{1}),\hat{B}(\nu_{2}),\ldots ,\hat{B}(\nu_{P})\right]$. According to (12), we have

(18) $$\begin{array}{c} {\hat{\mu }}_{p}^{d}=\underset{\mu_{p}^{\left(l\right)}}{\text{argmin}}\left[a{\left(\mu_{p}\right)}^{H}\hat{B}\left(\nu_{p}\right)a\left(\mu_{p}\right)\right]. \end{array}$$

Searching *μ*_*p*_ ∈ *μ*_*p*_^(*l*)^, the *P* largest peaks $\left({\hat{\mu }}_{1}^{d},{\hat{\mu }}_{2}^{d},\ldots ,{\hat{\mu }}_{P}^{d}\right)$ which correspond to the transmit angle can be obtained. Then the transmit angle *θ*_*p*_ can be expressed as ${\hat{\theta }}_{p}=a\sin({\hat{\mu }}_{p}^{d})*180/\pi$.

The main steps of Reduced-Dimension ESPRIT-MUSIC algorithm for angle estimation in the bistatic MIMO radar with sparse linear arrays as follows.

*Step  1.* Perform eigen-decomposition operation for the covariance matrix $\hat{R}$ to obtain **E**_*s*_ and **E**_*n*_.

*Step  2.* Calculate the ambiguous angle cosine estimates ${\hat{\mu }}_{p}^{a}$ and ${\hat{\nu }}_{p}^{a}$ via (8).

*Step  3.* Find the set of cyclically related candidates *μ*_*p*_^(*l*)^ and *ν*_*p*_^(*l*)^ by (9).

*Step  4.* Searching *ν*_*p*_ ∈ *ν*_*p*_^(*l*)^ with respect to (17), the *P* largest peaks $\left({\hat{\nu }}_{1}^{d},{\hat{\nu }}_{2}^{d},\ldots ,{\hat{\nu }}_{P}^{d}\right)$ can be found. Then compute the receive angle *φ*_*p*_.

*Step  5.* Searching *μ*_*p*_ ∈ *μ*_*p*_^(*l*)^ with respect to (18), the *P* largest peaks $\left({\hat{\mu }}_{1}^{d},{\hat{\mu }}_{2}^{d},\ldots ,{\hat{\mu }}_{P}^{d}\right)$ can be obtained. Then calculate the transmit angle *θ*_*p*_.

### 4.2. Simulation Results

**Figure 6:**
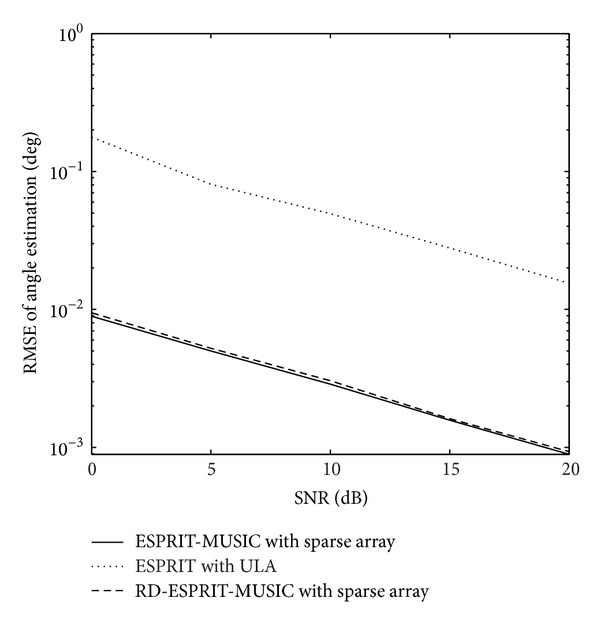
Angle estimation performance comparison.

**Figure 7:**
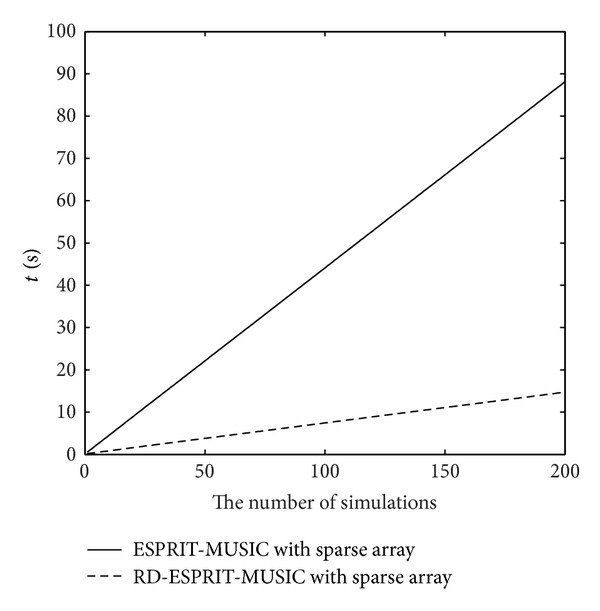
The runtime of algorithms comparison.

All simulations are implemented in the computer that consists of an Intel Core i3-2100 CPU at 3.10 GHz and an internal memory with capacity of 2 GB. Some simulations verify the angle estimation performance of RD-ESPRIT-MUSIC algorithm. To ensure fair comparison, all the experimental parameters are similar to Section 3.2. Assume that there exist *P* = 3 uncorrelated stationary targets, which are located at angles (*θ*_1_, *φ*_1_) = (15°, 20°), (*θ*_2_, *φ*_2_) = (25°, 40°), and (*θ*_3_, *φ*_3_) = (10°, 30°), and the number of snapshots is *L* = 100 for an *M*_*t*_ = 12, *M*_*st*_ = 3, *N*_*st*_ = 4 and *M*_*r*_ = 10, *M*_*sr*_ = 2, *N*_*sr*_ = 5 bistatic MIMO radar. And the intersubarray spacing *d*_*s*_ = 30∗(*λ*/2). From Figure 6, we can see that the angle estimation performance of RD-ESPRIT-MUSIC algorithm is similar to the ESPRIT-MUSIC algorithm. And all of them are much better than ESPRIT with ULA.  Figure 7 depicts the runtime comparison between ESPRIT-MUSIC algorithm and RD-ESPRIT-MUSIC algorithm. It is clearly shown that RD-ESPRIT-MUSIC algorithm pay much less time than ESPRIT-MUSIC algorithm. The simulation result demonstrates that the complexity of RD-ESPRIT-MUSIC algorithm reduces sharply.

## 5. Conclusion

In this paper, the ESPRIT-MUSIC method is proposed to estimate target angle in bistatic MIMO radar spaced sparse linear array. The proposed method can obtain the paired transmit angle and receive angle and avoid the manifold ambiguity. In order to reduce the computational complexity of the algorithm, we derive the RD-ESPRIT-MUSIC algorithm which only requires one-dimension search. All of them have much better performance than ESPRIT with ULA. Several simulation results are presented to verify the effectiveness of the two algorithms.
